# Challenges and opportunities in estimating viral genetic diversity from next-generation sequencing data

**DOI:** 10.3389/fmicb.2012.00329

**Published:** 2012-09-11

**Authors:** Niko Beerenwinkel, Huldrych F. Günthard, Volker Roth, Karin J. Metzner

**Affiliations:** ^1^Department of Biosystems Science and Engineering, ETH ZurichBasel, Switzerland; ^2^Swiss Institute of BioinformaticsBasel, Switzerland; ^3^Division of Infectious Diseases and Hospital Epidemiology, University Hospital Zurich, University of ZurichZurich, Switzerland; ^4^Department of Mathematics and Computer Science, University of BaselBasel, Switzerland

**Keywords:** next-generation sequencing, viral diversity, viral quasispecies, statistics, bioinformatics, haplotype inference, error correction, quasispecies assembly

## Abstract

Many viruses, including the clinically relevant RNA viruses HIV (human immunodeficiency virus) and HCV (hepatitis C virus), exist in large populations and display high genetic heterogeneity within and between infected hosts. Assessing intra-patient viral genetic diversity is essential for understanding the evolutionary dynamics of viruses, for designing effective vaccines, and for the success of antiviral therapy. Next-generation sequencing (NGS) technologies allow the rapid and cost-effective acquisition of thousands to millions of short DNA sequences from a single sample. However, this approach entails several challenges in experimental design and computational data analysis. Here, we review the entire process of inferring viral diversity from sample collection to computing measures of genetic diversity. We discuss sample preparation, including reverse transcription and amplification, and the effect of experimental conditions on diversity estimates due to *in vitro* base substitutions, insertions, deletions, and recombination. The use of different NGS platforms and their sequencing error profiles are compared in the context of various applications of diversity estimation, ranging from the detection of single nucleotide variants (SNVs) to the reconstruction of whole-genome haplotypes. We describe the statistical and computational challenges arising from these technical artifacts, and we review existing approaches, including available software, for their solution. Finally, we discuss open problems, and highlight successful biomedical applications and potential future clinical use of NGS to estimate viral diversity.

## Introduction

Many viruses, in particular RNA or single-stranded DNA viruses, exhibit extreme evolutionary dynamics. They have very high mutation rates, up to six orders of magnitude higher than in humans, short generation times, and large population sizes (Duffy et al., 2008). Under these conditions, genetic variants are produced constantly, and in each infected host, the virus population displays a high degree of genetic diversity. Rapidly evolving viruses are not only ideal systems for studying evolutionary mechanisms (Drummond et al., 2003), but many of them are significant pathogens of vital medical interest, including HIV, HCV, and Influenza (WHO, 2012).

Because of their diversity, intra-host virus populations are often referred to as mutant clouds, swarms, or viral quasispecies. The latter terms were originally introduced in the context of self-replicating macromolecules (Eigen, 1971; Eigen and Schuster, 1977) and have a precise mathematical meaning. A quasispecies is the equilibrium distribution of mutants in a mathematical model that accounts for mutation and selection (Eigen et al., 1988, 1989). In the framework of classical population genetics, it can be regarded as a coupled mutation-selection balance (Wilke, 2005). The main prediction of the quasispecies model is that selection acts on the population as a whole and hence the population dynamics cannot be understood from the fittest strain alone (Van Nimwegen et al., 1999; Wilke et al., 2001). The quasispecies model has later been applied to RNA viruses (Nowak, 1992; Domingo and Holland, 1997), hence the term viral quasispecies. The impact of the quasispecies model is not only due to its mathematical feasibility, but also its conceptual focus on the population as the target of natural selection (Burch and Chao, 2000).

The diversity of virus populations has repeatedly been shown to provide a selective advantage. For example, decreasing the mutation rate of poliovirus artificially, while maintaining its replication rate, resulted in reduced genomic diversity and in failure to adapt to adverse growth conditions (Vignuzzi et al., 2006). Similarly, pre-existing minority drug-resistant variants of HIV-1 have been shown to facilitate rapid viral adaptation leading to failure of antiretroviral therapy (Metzner et al., 2009; Li et al., 2011). In general, viral diversity is advantageous when the virus faces different selection pressures that need to be overcome by evolutionary escape (Iwasa et al., 2003, 2004). Changing selection pressures are common in the life of viruses, for example, after infecting a new host with a different immune response (Pybus and Rambaut, 2009), when infecting different cell types, while being exposed to different chemical agents, or due to changing multiplicity of infection (Ojosnegros et al., 2010). Understanding and modeling the escape dynamics of these processes is of direct relevance for clinical and public health decisions.

With the introduction of next-generation sequencing (NGS) technologies, the experimental analysis of viral genetic diversity has changed dramatically. Rather than using labor-intensive limiting dilution and individual cloning of viruses followed by traditional Sanger sequencing, NGS now allows for sampling the virus population in a highly parallel fashion in a single experiment. However, the novel high-throughput approach has several pitfalls associated with both the experimental protocol and the statistical analysis of the data. We address both aspects in this review and discuss several successful applications of NGS to viral diversity studies, including drug resistance, immune escape, and epidemiology.

## Sample preparation

**Figure 1:**
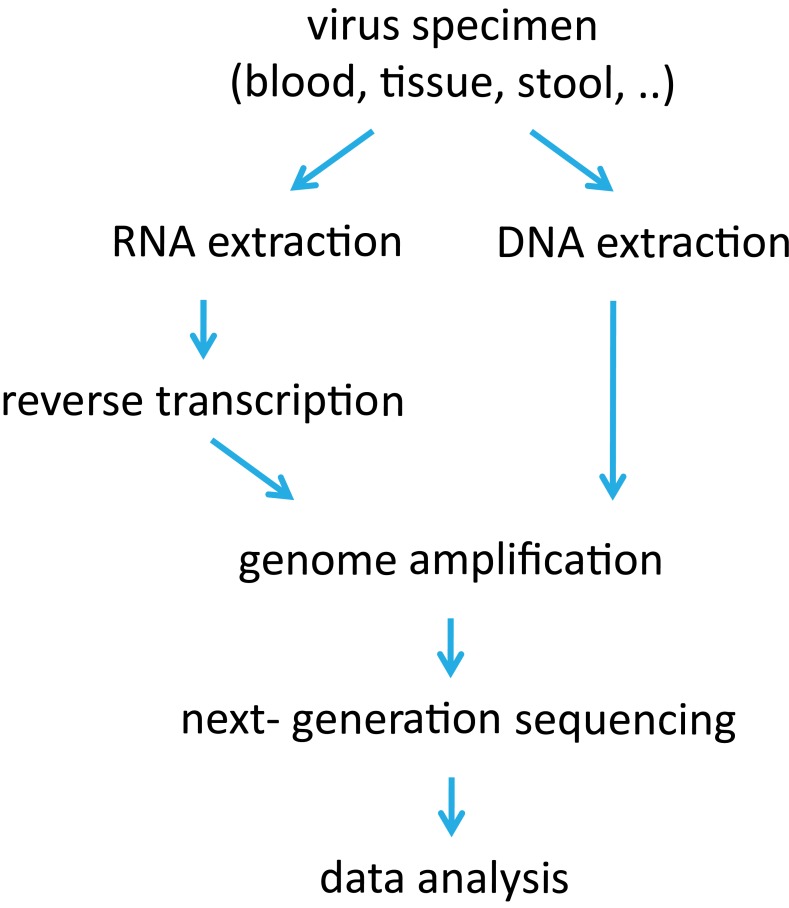
**Flow chart of sample processing for next-generation sequencing (NGS) of virus samples**.

The usefulness of NGS for viral diversity estimation depends crucially on the quality of the sample and on the procedure to prepare the sample. NGS sequence reads mirror the accumulation of errors, some of them preventable others unavoidable. To minimize the error rate, each step requires careful handling, starting with biological sample retrieval and storage up to the last steps of the NGS procedure itself (Figure 1).

Viral genomes are usually protected by the viral capsid and some of them additionally by an envelope, for instance, HIV and HCV. However, retrieval and storage conditions of biological specimens are especially important when studying RNA viruses due to the fragility of RNA (Holodniy et al., 1995; Jose et al., 2005), because degraded RNA will jeopardize all further steps of the analysis. Before starting the extraction of viral genomes, the viral load of the specimen should be considered. The final number of genome copies sequenced provides the basis for assessing viral diversity from the sequence reads (Metzner et al., 2003; Casbon et al., 2011). Low amounts might require a concentrating step, for instance, ultracentrifugation of plasma.

The choice of protocols used for genome extraction and elimination of contaminating RNA and DNA from other sources like host cells depends on the intended downstream procedures. Numerous kits are offered to extract viral DNA or RNA whose pros and cons will not be discussed here. A more critical point is the enrichment of viral genomes in the context of sample complexity. Three scenarios can be envisioned. (1) The virus is known and an amplicon approach is chosen for NGS. Here, the specificity of the primers might allow for amplifying the viral genome without any upstream enrichment. Nevertheless, it is often beneficial to eliminate contaminating DNA or RNA by DNase or RNase treatment. For instance, investigating HIV RNA genomes requires the elimination of proviral DNA genomes (Fischer et al., 2002). (2) The virus is known, but a random approach is chosen for NGS. Due to the high heterogeneity of some viruses, it might be disadvantageous to use virus-specific primers for amplification due to potential primer bias or even complete failure of amplification (Metzner et al., 2003). In contrast, any random approach, including amplification using degenerated or random primers as well as non-specific adaptor ligation and subsequent amplification using adaptor-specific primers, cannot differentiate between the viral genome and any other nucleic acid (Reyes and Kim, 1991; Chang et al., 1992). Thus, the elimination of contaminating nucleic acids is mandatory when a high coverage of viral genomes is required, as for studying diversity, since the viral genomes represent only a low-abundant fraction in almost all biological specimens (Daly et al., 2011). DNase and RNase treatment, filtration, density gradient centrifugation, and their combinations are commonly used procedures. Enrichment strategies based on hybridization capture might also be suitable (Turner et al., 2009; Althaus et al., 2012) and, potentially, freeze thaw nuclease digestion protocols may also be beneficial to minimize contaminating RNA or DNA (Fischer et al., 2002) (3). The virus in unknown, therefore, random approaches have to be applied. The enrichment of viral genomes is an even greater challenge in this set-up. In this review, we focus on estimating viral diversity from NGS data, a second step after virus discovery (Lipkin, 2010).

After viral genome extraction, an amplification procedure has to be performed, because the current NGS technologies require a high input DNA amount and the viral genome amount is several orders of magnitude lower. Furthermore, RNA genomes have to be reverse transcribed prior to PCR. Every amplification process introduces errors. Reverse transcriptases (RTs) are error-prone enzymes, because of the lack of any proof-reading activity (Preston et al., 1988; Roberts et al., 1988). Some RTs are less error-prone than others, but, in general, RT errors are unavoidable and very difficult to distinguish from real mutations since they are introduced in the first step of amplification. Another important but often ignored problem with reverse transcription is that short, incomplete cDNA fragments can act as primers in subsequent PCRs and lead to *in vitro* recombination. This phenomenon has been considered only for RT-PCRs amplifying several kilobases (kb) long fragments (Fang et al., 1998). We have recently shown that this effect also occurs very frequently when amplifying short cDNA fragments of a size of only 0.6 kb and can be minimized by using an RNaseH-negative RT (Di Giallonardo et al., submitted).

Four main types of errors can occur during PCR and are relevant for NGS data: (i) biased amplification due to primer mismatches, (ii) *in vitro* recombination due to premature termination of strand elongation and subsequent false hybridization of short DNA fragments acting as primers or, less frequently, due to template switching, (iii) nucleotide misincorporation due to the inaccuracy of DNA polymerases, and (iv) resampling due to, for instance, too low amounts of input DNA copies (Eckert and Kunkel, 1991; Liu et al., 1996; Kanagawa, 2003). Several precautions can be taken to minimize these errors. Primer mismatches can be diminished by choosing primer binding sites in conserved regions of the viral genome or by using degenerated primers. Chimera formation can be reduced by several improvements of PCR conditions such as increasing the elongation time, decreasing the number of cycles, and deleting the final extension step (Meyerhans et al., 1990; Judo et al., 1998). Nucleotide misincorporation can be lowered by using high-fidelity DNA polymerases, and resampling can be reduced, for instance, by optimizing the input copy number. Even when applying all these precautions, it is currently not possible to completely avoid these PCR errors. Furthermore, the discrimination between artificial and real viral variants can be very difficult if not impossible. One possibility is to perform several independent PCRs assuming that most of the errors occur randomly with regard to the sequence position and the timing of the error, i.e., in which PCR cycle the error occurs, resulting in different variants of different frequencies in the replicates. A recently described method uses primer identifiers (IDs) to uniquely label each cDNA molecule (Jabara et al., 2011). This is an elegant procedure to reduce or even eliminate PCR errors, although errors induced during the reverse transcription cannot be addressed in this manner. In addition, the method is only applicable to amplicon-based approaches and a high number of sequence reads are required to obtain a sufficient number of consensus sequences, each of which has to be derived from at least three reads with the same primer ID. Thus, all unique or twice occurring reads, which represent the majority of sequence reads, cannot be considered in the analysis.

Overall, sample preparation is a critical issue in the process of NGS. If unrecognized, errors during sample preparation can lead to an artificially increased diversity of the investigated virus population. To avoid such misinterpretation, the pitfalls of sample preparation need to be identified and properly addressed.

## Next-generation sequencing

In the last decade, many NGS technologies have been developed and several are commercially available today or about to become available in the near future (Mardis, 2008b; Metzker, 2010). Due to its massively parallel approach, NGS allows for generating much larger volumes of sequencing data in a cost-effective manner as compared to conventional sequencing methods. The increase in throughput has been so far-reaching that NGS is considered revolutionary, because it facilitates many new sequencing applications that had been out of reach (Mardis, 2008a; Schuster, 2008). One of these novel applications is the inference of viral genetic diversity from a single deep-coverage NGS experiment.

All NGS technologies involve the steps of template preparation, sequencing, and imaging, followed by data analysis, but they differ in the realization of each step. 454/Roche pyrosequencing has been the first NGS method commercially available and until today it is the most commonly used technology for the analysis of viruses (Margulies et al., 2005). For pyrosequencing, DNA is isolated, amplified and/or fragmented, adaptor-annealed, and amplified on beads in a micro-droplet emulsion PCR. DNA and beads have to be used in a ratio allowing the hybridization of only one DNA molecule to one bead, i.e., the majority of beads do not contain any DNA molecule. Thus, on each DNA-hybridized bead, a single template gives rise to several thousand copies. These beads are separated from the empty beads and loaded into 1.6 million wells of a picotiter plate, one bead per well, and enzymes for pyrophosphate sequencing are added. Sequencing by synthesis proceeds by adding the four bases in a cyclic order. In each cycle, the light emission associated with base incorporation is detected and remaining chemicals are washed out. The intensity of the light signal is approximately proportional to the number of nucleotides that have been incorporated. All generated signals are recorded as a series of peaks, called a flowgram, from which DNA bases are eventually called (Margulies et al., 2005).

The Illumina Genome Analyzer and HiSeq systems are currently dominating the NGS market (Bentley et al., 2008). Rather than emulsion PCR, Illumina relies on solid-phase amplification, which consists of initial priming and extending of single-stranded templates, followed by bridge amplification of each immobilized template with adjacent primers. In multiple cycles of annealing, extension, and denaturation, around 200 million molecular clusters are formed. For sequencing, all four nucleotides are added simultaneously. Each nucleotide is labeled with a different dye and they are modified to terminate DNA synthesis after incorporation. Color imaging is used to detect the incorporated nucleotide. In a cleavage step, the fluorescent dye is removed and termination is reversed by regenerating the 3′-OH group. Bases are called from the resulting four-color images.

We focus here on the 454/Roche and Illumina platforms, because the vast majority of reported virus sequencing applications have used these systems, but several other technologies can, and are likely to, be used as well, including ABI SOLiD, Ion Torrent, PacBio RS, and Polonator. The technical details in which platforms differ can have important consequences for their applicability to viral sequencing studies. Among other aspects, NGS platforms differ in throughput, runtime, costs, read lengths, and error patterns (Metzker, 2010). The currently most powerful 454/Roche sequencer GS FLX Titanium XL+ can produce up to 1 million reads per run of 700 bp average length, while Illumina's largest machine, HiSeq 2500, can generate up to 1.2 billion paired-end reads of 2 × 150 bp length. Both companies also offer smaller benchtop devices of their platforms that may be preferable in certain diagnostic and clinical settings. The Roche/454 Junior produces up to 100,000 reads of 400 bp average length in a single 10-h run, and the Illumina MiSeq generates up to 30 million paired-end reads of 2 × 150 bp length in 24 h. Thus, longer reads can be produced with the 454/Roche technology, but ultra-deep coverage is easier to obtain with Illumina (Loman et al., 2012).

In addition to the various errors that can occur during sample preparation, as discussed in “Sample Preparation”, all NGS platforms introduce sequencing errors. With 454/Roche pyrosequencing, insertions and deletions (indels) are the most common type of errors. They occur predominantly in homopolymeric regions of the target sequence, where the linear relationship between signal intensity and number of incorporated nucleotides starts to fail. Remaining nucleotides after washing can give rise to insertions or carry forward errors, while deletion errors can result from incomplete extension (Margulies et al., 2005; Balzer et al., 2011). The error rate has been shown to increase with read length and to depend on several other biological and technical factors, including the organism and genomic region to be analyzed and the position on the picotiter plate with respect to the flow of chemicals and the position of the camera (Gilles et al., 2011).

Illumina reads are not as susceptible to indel errors in homopolymeric regions, but artificial indels outside these regions and substitutions have similar frequencies (Archer et al., 2012). The Illumina mismatch rate also increases with read length and it further depends on the sequence context and the substitution type (Dohm et al., 2008; Kircher et al., 2009; Nakamura et al., 2011). Illumina reads are generated in forward and reverse direction, and errors predominantly occur on one of the two strands (Chapman et al., 2011; Varela et al., 2011). All NGS platforms report quality scores, defined as *Q* = −10log_10_ *p*, where *p* is the error probability (Ewing and Green, 1998), together with the called bases, but the calibration of these scores is challenging (Brockman et al., 2008; Kircher et al., 2009) and there is no consensus on how to compare scores across platforms.

Besides errors, the distribution of reads along the genome is critical for diversity estimation, especially if phasing of genetic variants is the goal. However, uniform coverage is difficult to achieve and, in practice, the read coverage often varies by orders of magnitude. The reasons for this variation are poorly understood, but for Illumina, the GC content of the target sequence is an important factor (Dohm et al., 2008). Uniform coverage is feasible within short segments by using a single amplicon. However, increasing the number of amplicons to cover longer segments can impair this uniformity, and shot-gun approaches introduce even more variation. For 454/Roche, Illumina, and ABI SOLiD, correlation of coverage and errors is fairly weak among the three different NGS platforms (Harismendy et al., 2009). Thus, for viral diversity estimation, where uniform coverage and error correction are critical, complementary sequencing strategies involving more than one platform may be more efficient than increasing the coverage on a single platform.

The large amounts of viral sequencing data obtained by NGS place substantial demands on information technology and computational data analysis in terms of storage, quality control, mapping, error correction, single nucleotide variant (SNV) calling, haplotype reconstruction, diversity estimation, and data integration (Pop and Salzberg, 2008; Vrancken et al., 2010; Barzon et al., 2011; Beerenwinkel and Zagordi, 2011). Data analysis usually starts by removing reads of exceptionally low quality. The rationale for this initial filtering step is that low-quality reads contribute disproportionally to the overall error rate, i.e., most errors occur on a few reads (Huse et al., 2007). Filtering can be based on quality scores or on properties of the read or the target sequence known to affect error rates, as discussed above. Optimized filtering has been shown to reduce the error rate in detecting genomic variation up to 300-fold (Reumers et al., 2011).

After filtering, the next step is to align the remaining reads. In re-sequencing studies of known viruses, this is typically done by mapping reads individually to a reference sequence and then aggregating the pairwise alignments into a multiple sequence alignment (MSA). For read mapping, local alignment using dynamic programming may be applied (Wang et al., 2007; Zagordi et al., 2011), but for larger data sets, efficient short read mappers are required. Several efficient mapping algorithms based on indexing techniques are available. Some of them can handle gaps, account for quality scores, and have a paired ends option (Trapnell and Salzberg, 2009; Wikipedia, 2012). In coding regions, a major goal of the alignment step is to identify indels that cause frameshifts. These alterations are likely to be sequencing errors, which are frequently observed using the 454/Roche platform. Hence, they are usually removed, but this bears the risk of losing virus variants harboring real indels. For correcting indel errors, a high-quality alignment is necessary, but in mixed samples, the use of a reference sequence can be suboptimal if reads originating from some subpopulations align only poorly to the reference sequence. To address this concern, a MSA may be computed directly, for example, by using a progressive MSA strategy that takes into account the approximate location of reads on the genome (Saeed et al., 2009). Similarly, for the HIV *env* gene, a multi-step procedure has been proposed, in which reads are located efficiently on a reference sequence by k-mer matching and MSAs are built locally in windows of width 70 nucleotides along the genome. From all local MSAs, in-frame consensus sequences are generated and concatenated. Finally, the reads are re-aligned to the global consensus sequence and all indels causing frameshifts are removed. Using the consensus rather than a reference sequence was shown to improve the alignment quality, especially if their divergence is high (Archer et al., 2010).

## Local diversity estimation

From the aligned reads, one wants to reconstruct the original virus population in the sample, meaning the composition and relative frequencies of all individual viral genomes, also referred to as strains or haplotypes. Even after filtering and removal of frameshift-causing indels, many reads are still erroneous. Therefore, in mixed samples, error correction and haplotype inference are intrinsically tied to each other and, in fact, addressed jointly by most computational methods. This is in contrast to the simpler task of error correction in clonal samples, where implausible variants can easily be discarded using either k-mers, suffix trees/arrays, or MSA (Yang et al., 2012).

**Figure 2:**
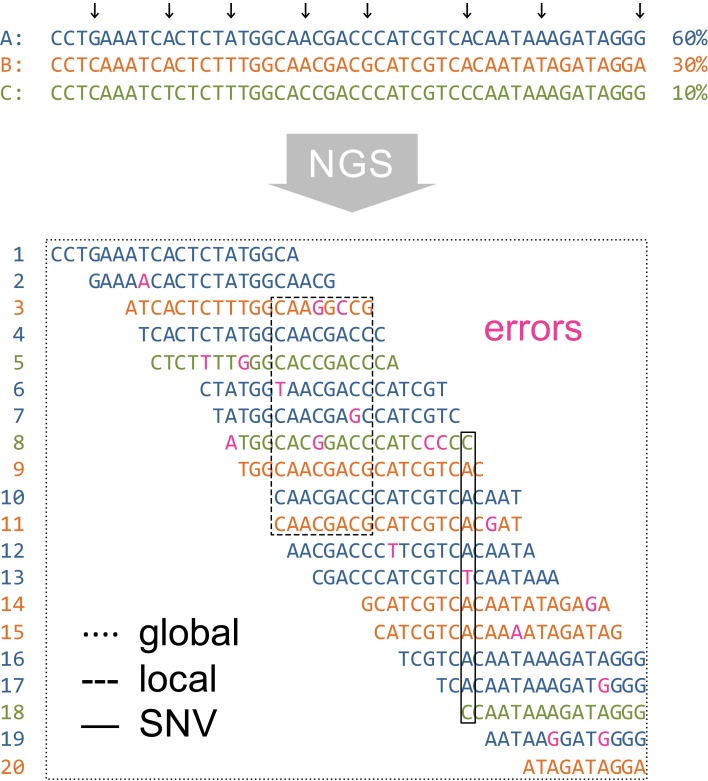
**Spatial scales of diversity estimation from NGS data.** In this example, it is assumed that the true virus population (top of figure) consists of three haplotypes of relative frequencies 60% (A, blue), 30% (B, orange), and 10% (C, green). Segregating sites are indicated by arrows. Twenty short reads (labeled 1 through 20) are generated by NGS from the virus population subject to sequencing errors (indicated in magenta). Reads are displayed in a MSA and in the color of their corresponding parental haplotype. Diversity estimation can be approached at single sites (SNV detection, solid-line rectangle), in windows of the MSA (local haplotype inference, dashed-line rectangle), or over the entire genomic region (global haplotype reconstruction, dotted-line rectangle).

The haplotype inference problem occurs at different spatial scales depending on the length of the genomic region to be analyzed for diversity (Figure 2). When only a single genomic site is considered, diversity estimation means detecting SNVs. Local haplotype inference refers to analyzing windows in the MSA that are covered entirely by reads. Finally, global haplotype inference, also called quasispecies assembly, involves a jigsaw puzzling step of assembling local fragments into multiple haplotype sequences that span the entire genomic region of interest.

SNV calling is based on the observed nucleotide counts at a single sequence position. The simplest statistical model for separating errors from true variations is to assume that, at each genomic site, the number of errors follows the same Poisson distribution and to call SNVs that occur more often than expected by chance for a given error rate (Wang et al., 2007). This approach has been extended to account for site-specific error rates (Macalalad et al., 2012). The power and accuracy of SNV calling can be increased substantially by a control experiment, in which the same genomic region is sequenced from a clonal sample under conditions as similar as possible to those for the mixed sample. The rationale for this comparative sequencing approach is that the control experiment allows for estimating the specific error patterns of the experiment and hence for improved separation of biological signal from technical noise. In this setting, SNV detection is based on comparing nucleotide counts between two experiments, for example, using Fisher's exact test (Koboldt et al., 2012). Assuming independent Poisson distributions, another test is based on the difference of the number of observed nucleotides (Altmann et al., 2011). Count data from NGS experiments have repeatedly been shown to display more variation across sites than is captured by a binomial distribution, and the beta-binomial distribution is a popular choice for such overdispersed data (Flaherty et al., 2012; Gerstung et al., 2012). Based on this model and accounting for the strand-bias of sequencing errors, a sensitivity of up to 1/10,000 has been achieved for SNV calling at a coverage of around 10^5^ (Gerstung et al., 2012).

By dropping the assumption of independence among sites, SNV calling can be further improved. Considering the number of joint sequencing errors at two positions has been shown to significantly decrease the minimal frequency at which a variant is detectable (Macalalad et al., 2012). This phasing of two SNVs is possible only at a distance smaller than the maximal read length. For small distances, the SNV pair will be covered by many reads, but for larger distances the benefit of phasing will be undone by the loss of joint coverage. In fact, for deep coverage, pairs are more informative than single sites only if their distance is not larger than the average read length (Macalalad et al., 2012).

**Figure 3:**
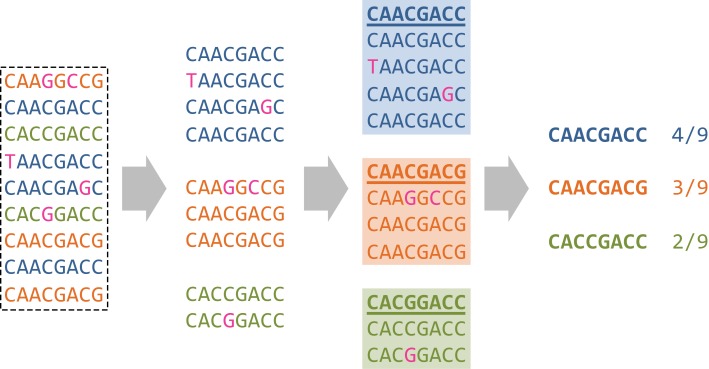
**Local read clustering.** The local window of the MSA displayed in Figure 2 is considered (dashed-line rectangle), with colors defined as in Figure 2. Reads that are more similar to each other than to other reads are grouped together which recovers the three original haplotypes A, B, and C of Figure 2 as indicated by the three different colors. Each cluster center sequence is a predicted haplotype (bold, underlined) and the size of its corresponding cluster is an estimate of the frequency of the haplotype (here, 4/f/9, and 2/9, respectively).

The idea of phasing SNVs is further extended by comparing entire reads within a sequence window they overlap. The size of the window is subject to the same trade-off as the distance between two SNVs discussed above: Small windows contain many reads but few SNVs for robust pairwise comparisons of reads, while large windows contain less reads but more segregating sites. Local haplotype inference is based on clustering reads within a given window (Figure 3). The rationale for clustering is that reads originating from the same haplotype should be more similar to each other than to reads from other haplotypes. This assumption is only valid if the error rate is low relative to the diversity of the population, and the ability to identify haplotype clusters increases with coverage (Eriksson et al., 2008).

Clustering was initially performed using the classical k-means algorithm (Jain and Dubes, 1981) and later formulated probabilistically and solved in a Bayesian fashion (Eriksson et al., 2008; Zagordi et al., 2010a). In particular, the latter approach allows for estimating the error rate and the number of clusters from the data—a notoriously difficult problem with any clustering method. The cluster centers are the predicted haplotypes and the cluster sizes are interpreted as the haplotype frequencies in the population. Error correction is based on a local read clustering solution by replacing all read bases with those of its cluster center (Figure 3). This method has been shown to reduce the per-base error rate after correction, to increase the sensitivity and specificity of local haplotype calling, and to improve the estimation of haplotype frequencies as compared to simple read counting or k-means clustering (Zagordi et al., 2010b). For the 454/Roche platform, a similar clustering approach called AmpliconNoise can be applied before base calling on the flowgrams (Quince et al., 2009, 2011). Here, the observed flowgrams are obtained from ideal flowgrams corresponding to read sequences subject to measurement noise. Whether clustering is based on sequences or on flowgrams, the distance measure between reads should reflect the pattern of experimental noise.

As an alternative to clustering, k-mer-based error correction, implemented in the program KEC, has been proposed for viral amplicon sequencing (Skums et al., 2012). This approach extends the EDAR error correction algorithm (Zhao et al., 2010) and initially does not require a read alignment. It consists of a number of heuristic steps with the goal of locating error regions in reads by considering rare k-mers and removing errors in these regions. In a final step, which eventually involves MSAs of the corrected reads, local haplotypes are reconstructed.

## Global diversity estimation

The local methods discussed in the previous section focus on reconstructing haplotypes in a local window, the maximum size of which is effectively restricted to the average length of the reads. The global reconstruction problem, on the other hand, is defined as the genome-wide assembly of quasispecies, irrespective of machine-specific parameters like the average read length. The various approaches to solving this jigsaw puzzle described in the literature can be roughly divided into three groups: (1) graph-based methods that first aggregate the reads in a read graph and then search for a minimum set of paths through this graph, (2) probabilistic clustering models based on mixture models, and (3) *de novo* assembly methods which do not rely on the availability of a reference sequence.

**Figure 4:**
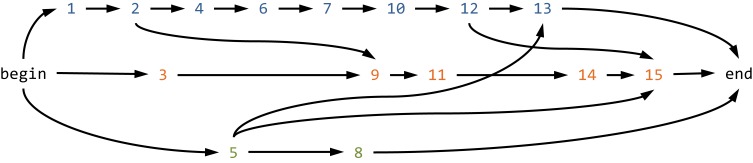
**Read graph-based global haplotype reconstruction.** Shown is the read graph for the first 15 reads of the MSA shown in Figure 2. Each read is represented by its index and colored according to its parental haplotype (A, blue, first row; B, orange, second row; and C, green, third row). Reads are connected by a direct edge if they agree on their non-empty overlap. Each path from the begin node to the end node represents a potential global haplotype, but there are more paths in the graph than the original three haplotypes the reads have been derived from.

Read graph-based global haplotype reconstruction consists in aggregating the reads in a read graph and subsequently identifying haplotypes as paths in this graph. The concept of a read graph has been independently introduced by Eriksson et al. (2008) and Westbrooks et al. (2008). The read graph contains the possibly pre-processed, for instance, locally error-corrected, reads as nodes. Directed edges connect two nodes when the reads agree on their non-empty overlap (Figure 4). The direction of the edge reflects the order of the starting positions on the reference sequence. The set of nodes is restricted to all irredundant reads, where a read is considered redundant if there is another read that overlaps completely and if both reads agree on this overlap. In a similar manner, the set of edges is restricted to include only those edges for which there would be no path between the corresponding nodes without this edge. The latter restriction is called transductive reduction in (Westbrooks et al., 2008), and it has been shown that this reduction can be computed efficiently. Finally, a source and a sink node are added to the graph, along with edges connecting all reads starting at the first position to the source and all reads ending at the last position to the sink (Figure 4).

Every path in the read graph connecting source and sink is a potential haplotype, and the problem of estimating the haplotypes present in a certain sample might be restated as finding a set of such source-sink paths that explains the reads well. Different formalizations of this problem lead to different optimization problems. One example is the search for the minimum set of paths that covers all reads implemented in ShoRAH (Eriksson et al., 2008; Zagordi et al., 2011). The same problem has been studied in a different way as a network flow problem (Westbrooks et al., 2008). A variant of the network flow formulation is the search for a set of haplotypes covering all reads with minimum costs (Westbrooks et al., 2008) and, in a slightly different fashion relaxing the requirement of a complete read cover, implemented in ViSpA (Astrovskaya et al., 2011). The combinatorial reconstruction is followed by frequency estimation using an Expectation Maximization (EM) algorithm (Eriksson et al., 2008; Westbrooks et al., 2008; Astrovskaya et al., 2011).

In a related approach termed QuRe, the same read graph idea is used to find a set of consistent quasispecies explaining the reads (Prosperi et al., 2011; Prosperi and Salemi, 2012). It differs from the methods above in the optimization procedure for finding the quasispecies. This is formalized as minimizing the number of *in silico* recombinants instead of finding a path cover explaining the reads. However, both optimization strategies are similar in nature, since avoiding *in silico* recombinants can be regarded as avoiding redundant paths in the read graph. Another advantage of QuRe is that it explicitly addresses the blockwise structure of the reads due to amplicon-based sequencing in the statistical analysis (Prosperi et al., 2011; Prosperi and Salemi, 2012).

Haplotype assembly based on amplicon sequencing is also addressed by the BIOA software (Mancuso et al., 2011). Here, a read graph-based framework is proposed that includes balancing of haplotype frequencies between neighboring amplicons followed by quasispecies reconstruction using a maximum bandwidth approach or a greedy algorithm. In the assembly step, the parsimony criterion of explaining the observed reads with a minimal number of haplotypes is relaxed to finding a quasispecies of minimal entropy explaining the reads. This strategy was shown to outperform shotgun-based quasispecies assembly using ViSpA.

QColors is another method that relies on the read graph as the main source of information for assembling reads into haplotypes, but it uses in addition a conflict graph consisting of edges between reads that overlap but disagree on the overlap (Huang et al., 2011). The reconstruction problem is then to find a partition of the reads into a minimal number of non-conflicting subsets, which defines a vertex graph coloring problem, hence the name QColors. A potential problem with this approach might be the sensitivity of the conflict graph to sequencing errors and the uncertainty in placing alignment gaps, which are not explicitly dealt with.

Another method that uses the read graph approach is called Hapler (O'Neil and Emrich, 2012). This method is specifically designed for situations characterized by low haplotype diversity and low read coverage (<25×), which, for instance, occur in the context of population-level *de novo* transcriptome assemblies or ecological studies. The minimum path cover problem is generalized and reformulated as a weighted bipartite graph matching problem, such that erroneous reads can be identified. Since, in general, the resulting path covers are again not unique, the analysis is equipped with a randomization step in which samples are drawn from the set of path covers, although this process seems to lack a clear probabilistic interpretation. Experiments under low-coverage conditions indicate that this method is successful in reconstructing local haplotypes over a region that is roughly determined by the average read length, which in our terminology would be classified as local reconstruction. Nevertheless, longer haplotype assemblies are possible with Hapler and specific care is taken in reconstructing consensus sequences with a minimal number of chimeric points.

A common property of all read graph-based approaches is that the haplotype reconstruction problem itself becomes deterministic in nature, while the unavoidable noise component present in observed reads is dealt with in a pre-processing error correction step—if at all.

Removing all the stochasticity in the observed reads by way of local error correction prior to global haplotype reconstruction has the limitation that corrections cannot be revised in the global context and miscorrections are propagated through subsequent steps. A probabilistic hierarchical model that circumvents this problem has been introduced (Jojic et al., 2008). The main idea is to model the generative stochastic process of read generation. Parameters and hidden variables in this method include the parental haplotype, the starting position, and the parameters related to the error transformation. Inference is carried out by maximizing the likelihood using the EM algorithm. A potential drawback of this approach is that the user has to fix the number of haplotypes to be reconstructed in advance, and no well-defined estimation process for this number is provided.

**Figure 5:**
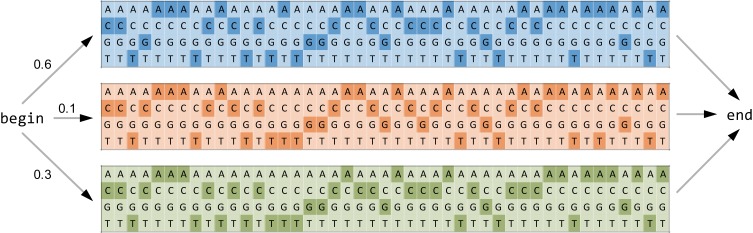
**Probabilistic global haplotype reconstruction using a generative mixture model.** Each of the three haplotypes colored as in Figure 2 (A, blue; B, orange; and C, green) is represented as a chain of probability tables over the four nucleotides, where darker shading of a base indicates higher probability. The probabilities of traversing from the begin node to one of the haplotypes serve as an estimate for the haplotype frequencies. Each read is regarded as an independent observation from this statistical model.

Probabilistic approaches are a second methodology for global haplotype reconstruction. PredictHaplo is one of these approaches which also automatically adjusts the number of haplotypes (Prabhakaran et al., 2010). In this model, a haplotype is represented as a set of position-specific probability tables over the four nucleotides, which can be augmented to include a fifth character representing alignment gaps (Figure 5). The underlying generative model assumes that reads are sampled from a mixture model, where each mixture component is interpreted as a haplotype, and the associated mixing proportion estimates the haplotype frequency. In order to avoid a priori specification of the number of mixture components, an infinite mixture model is employed (Ewens, 1972; Ferguson, 1973; Rasmussen, 2000), and for computational reasons, a truncated approximation of this stochastic process is used.

A further refinement of probabilistic haplotype reconstruction has been implemented in the program QuasiRecomb (Zagordi et al., 2012). Here, haplotypes are not reconstructed individually, but rather their distribution is estimated by a hidden Markov model. The model assumes that all haplotypes are generated from a small set of sequences by mutation and recombination. This model is taking into account that in some RNA viruses, such as HIV, recombination is very frequent and hence an important factor generating genetic diversity.

All approaches described so far make use of a known reference genome that serves as a fixed spatial coordinate system after read alignment. By contrast, *de novo* assembly methods are more general in nature since they do not require such reference genomes. Several assemblers specifically designed for certain NGS platforms like 454/Roche have been proposed in recent years (Finotello et al., 2012). The original goal of *de novo* assembly is reconstructing a single target genome sequence, rather than an ensemble of different genomes. Hence, the currently available genome assemblers are not designed to solve the whole-genome quasispecies assembly problem, but the different contigs they reconstruct may serve as a starting point for this jigsaw puzzle (Ramakrishnan et al., 2009).

Large-scale simulation studies show that all global reconstruction methods rely on the availability of relatively long reads. Coverage is also important when it comes to detecting low-abundant mutants, but even an arbitrarily high coverage cannot compensate for insufficient overlaps due to short reads. Given the typical diversity of virus populations, it appears that global haplotype reconstruction is currently only realistic for sequencing platforms producing long reads on the order of at least 300–500 bp. Accordingly, successful reconstructions have been reported predominantly for the 454/Roche sequencing platform.

Regarding the different computational approaches described above, it is generally difficult to conduct informative comparative simulation experiments, but two general trends have become evident. First, local read error correction has the tendency to under-correct the reads, which can lead to a large number of false positive global haplotypes, in particular, when combined with read graph approaches requiring a complete coverage of all reads. Quasispecies assembly methods that relax this coverage requirement (Astrovskaya et al., 2011; O'Neil and Emrich, 2012) or probabilistic approaches avoiding the read-graph construction (Jojic et al., 2008; Prabhakaran et al., 2010) are successful in decreasing the false positive rate. Second, the most problematic step in genome-wide reconstruction is the usually unavoidable (RT-)PCR pre-processing which can introduce significant artifacts. These artifacts might have a much stronger effect on the final quality of the haplotype reconstruction than the actual choice of the computational reconstruction method.

Computational methods for local and global haplotype reconstruction are summarized in Table 1. All of these tools have been developed in research environments and most are subject to continuous enhancements. Their usability and performance also depends on the quickly changing characteristics of the sequencing machines. In the future, comparative studies using simulated data, mixed control samples, or Sanger-sequenced gold standard samples are required to assess the performance of these tools under different conditions. In addition, software tools are available for NGS read data management and visualization. For example, Segminator II has been specifically designed to display sequence variability of temporally sampled virus populations (Archer et al., 2012).

**Table 1:** **Available software tools for viral quasispecies inference**.

**Program**	**Method**	**URL**	**References**
QuRe	Read graph	http://sourceforge.net/projects/qure/	Prosperi and Salemi, 2012
ShoRAH	Read graph	http://www.cbg.ethz.ch/software/shorah	Zagordi et al., 2011
ViSpA	Read graph	http://alla.cs.gsu.edu/~software/VISPA/vispa.html	Astrovskaya et al., 2011
BIOA	Read graph	https://bitbucket.org/nmancuso/bioa/	Mancuso et al., 2011
Hapler	Read graph	http://nd.edu/~biocmp/hapler/	O'Neil and Emrich, 2012
AmpliconNoise	Probabilistic	http://code.google.com/p/ampliconnoise	Quince et al., 2011
PredictHaplo	Probabilistic	http://www.cs.unibas.ch/personen/roth_volker/HivHaploTyper	Prabhakaran et al., 2010
QuasiRecomb	Probabilistic	http://www.cbg.ethz.ch/software/quasirecomb	Zagordi et al., 2012

## Applications

NGS is widely applied to study viral diversity mainly in the context of drug resistance of clinically relevant viruses such as HIV, HCV, and HBV (Table 2). Most studies focus on pre-existing minority drug-resistant virus variants in treatment-naïve individuals and their impact on the success of antiviral therapy, epidemiological surveillance, and virus population dynamics during virological failure. The pathways of drug resistance development are of particular clinical importance, since they can lead to new drug design or new therapeutic strategies, for instance, avoiding cross resistance or rapid selection of resistant viruses (Beerenwinkel et al., 2003). Furthermore, epidemiological studies for a huge variety of human pathogenic viruses were performed using NGS technologies, including cytomegalovirus (CMV), Epstein Barr virus (EBV), HCV, influenza virus, norovirus, rhinovirus, rotavirus, and varicella zoster virus (VZV) (Table 2).

**Table 2:** **Applications of 454/Roche pyrosequencing and Illumina NGS technologies in clinical virology**.

**Virus**	**Study**	**NGS platform**	**NGS approach**	**Basis of analysis**	**References**
CMV	Epidemiology	454/Roche	Amplicon-based	Reads	Gorzer et al., 2010
CMV	Epidemiology	454/Roche	Shotgun	Consensus sequence	Jung et al., 2011
EBV	Epidemiology	Illumina	Shotgun	SNV, consensus sequence	Liu et al., 2011
EBV	Epidemiology	Illumina	Shotgun (amplicons)	SNV	Kwok et al., 2012
HBV	Drug resistance	454/Roche	Amplicon-based	Reads, SNV	Solmone et al., 2009; Homs et al., 2011; Rodriguez-Frías et al., 2012
HBV	Drug resistance	454/Roche	Amplicon-based	SNV	Margeridon-Thermet et al., 2009; Ko et al., 2012; Sede et al., 2012
HBV	Drug resistance	Illumina	Shotgun	SNV	Nishijima et al., 2012
HCV	Drug resistance	454/Roche	Amplicon-based	Reads	Bolcic et al., 2012; Fonseca-Coronado et al., 2012
HCV	Drug resistance	Illumina	Shotgun (cDNA)	SNV	Hiraga et al., 2011
HCV	Drug resistance	454/Roche	Shotgun (amplicons)	SNV, consensus sequences	Lauck et al., 2012
HCV	Drug resistance	Illumina	Paired-end (amplicons)	SNV	Nasu et al., 2011
HCV	Drug resistance	454/Roche	Amplicon-based	SNV	Powdrill et al., 2011
HCV	Epidemiology	454/Roche	Amplicon-based	Reads	Escobar-Gutiérrez et al., 2012; Forbi et al., 2012
HCV	Epidemiology	Illumina	Shotgun (cDNA)	SNV, consensus sequences	Ninomiya et al., 2012
HIV	Drug resistance	454/Roche	Amplicon-based	SNV	Hoffmann et al., 2007; Wang et al., 2007; Mitsuya et al., 2008; Le et al., 2009; Simen et al., 2009; Varghese et al., 2009; Lataillade et al., 2010, 2012; Alteri et al., 2011; D'Aquila et al., 2011; Delobel et al., 2011; Gianella et al., 2011; Ji et al., 2011; Kozal et al., 2011; Moorthy et al., 2011; Stelzl et al., 2011; Fisher et al., 2012; Messiaen et al., 2012
HIV	Drug resistance	454/Roche	Amplicon-based	Reads, SNV	Hedskog et al., 2010; Ji et al., 2010; Mild et al., 2011; Mukherjee et al., 2011; Armenia et al., 2012
HIV	Epidemiology	454/Roche	Shotgun (amplicons)	Consensus sequence	Bruselles et al., 2009
HIV	Epidemiology	454/Roche	Amplicon-based	Consensus sequence	Eshleman et al., 2011
HIV	Epidemiology	454/Roche	Amplicon-based	Reads	Redd et al., 2012
HIV	Tropism	454/Roche	Amplicon-based	Reads	Archer et al., 2009; Rozera et al., 2009; Abbate et al., 2011; Swenson et al., 2010; Vandenbroucke et al., 2010; Baatz et al., 2011; Bunnik et al., 2011; Raymond et al., 2011; Saliou et al., 2011; Svicher et al., 2011; Swenson et al., 2011a,b; Vandekerckhove et al., 2011
Influenza A virus	Epidemiology	Illumina	Shotgun (amplicons)	SNV	Kuroda et al., 2010; Kampmann et al., 2011
Influenza A virus	Epidemiology	454/Roche	Shotgun (amplicons)	SNV	Bartolini et al., 2011
Influenza A virus	Epidemiology	454/Roche	Shotgun	Reads	Lorusso et al., 2011
norovirus	Epidemiology	454/Roche	Shotgun (amplicons)	SNV, haplotype recon-struction	Bull et al., 2012
rhinovirus	Epidemiology	Illumina	Shotgun (amplicons)	SNV, consensus sequences	Tapparel et al., 2011
rotavirus	Epidemiology	454/Roche	Shotgun (cDNA)	Consensus sequences	Jere et al., 2011
VZV	Epidemiology	454/Roche	Shotgun (amplicons)	Consensus sequences	Zell et al., 2012

BAL, bronchoalveolar lavage; CMV, cytomegalovirus; EBV, Epstein Barr virus; HBV, hepatitis B virus; HCV, hepatitis C virus; HIV, human immunodeficiency virus; SNV, single nucleotide variant; VZV, varicella zoster virus.

NGS is also increasingly used in more basic research areas, such as characterization of transmitted HIV (Fischer et al., 2010) and HCV (Wang et al., 2010; Bull et al., 2011), estimation of infection dates (Poon et al., 2011), evolution during the course of infection with HIV (Rozera et al., 2009; Poon et al., 2010; Wu et al., 2011), HCV (Bull et al., 2011), and rhinovirus (Cordey et al., 2010), and hypermutation patterns (Reuman et al., 2010; Knoepfel et al., 2011). Recently, NGS technologies have been applied to obtain the whole genome of HIV using a coverage allowing quasispecies analysis beyond the generation of consensus sequences to study, for instance, patterns of immune escape (Bimber et al., 2010; Willerth et al., 2010; Henn et al., 2012).

All these applications demonstrate the growing importance of NGS in studying viral diversity. With this technology, we will gain further insights into transmission traits, viral evolution, and its association with pathogenesis. World-wide viral diversity surveillance will be important for vaccine design and vaccination strategies. Currently, genetic diversity is mainly studied based on the detection and analyses of SNVs, rather than the reconstruction of linked mutations, due to the challenges in local and global haplotype reconstruction discussed above. It will be a huge step forward when haplotype reconstruction in heterogeneous viruses matures into a routine procedure based on standardized experimental protocols and validated, automatic data analysis pipelines.

## Outlook and conclusions

NGS opens up new roads to study viral diversity. It will tremendously increase our knowledge in virus evolution, fitness, selection pathways, and pathogenesis. Together with host genomics, viral diversity will allow insights into complex virus-host interactions. Full-length viral sequences may ultimately define truly conserved regions in viral genomes which might also be of relevance for vaccine and drug design. Clinically, the first application we can foresee is that in a single assay all drug targets relevant for antiviral treatment can be sequenced including information on minority drug-resistant variants. For all applications, sample procedures have to be chosen that minimize errors during sample preparation and sequencing. Several challenges in data analysis remain, especially in regard to alignments and global diversity estimation. In the future, some of these challenges might be diminished by upcoming third- and fourth-generation sequencing technologies, like single molecule or direct RNA sequencing.

Another not yet addressed future challenge will be making sense of the large amounts of genome data generated by NGS. For instance, clinical cut-offs need to be defined for minority drug-resistant virus variants, the clinical importance of new virus subtypes or even new viruses needs to be determined, and pathogenesis factors need to be confirmed in clinical settings. Thus, downstream analyses have to include large sets of well-documented patients, results from other experimental set-ups, etc. These are challenges as well as opportunities to answer important research questions which could not be addressed with conventional sequencing techniques.

### Conflict of interest statement

Karin J. Metzner has received travel grants and honoraria from Gilead, Roche Diagnostics, GlaxoSmithKline, Bristol-Myers Squibb, Tibotec, and Abbott, and has received research grants from Abbott, Gilead, and Roche Diagnostics. Huldrych F. Günthard has been an adviser and/or consultant for the following companies: GlaxoSmithKline, Abbott, Novartis, Gilead, Boehringer Ingelheim, Roche, Tibotec and Bristol-Myers Squibb, and has received unrestricted research and educational grants from Roche, Abbott, Bristol-Myers Squibb, GlaxoSmithKline, Gilead, Tibotec and Merck Sharp & Dohme (all money went to institution). The other authors declare that the research was conducted in the absence of any commercial or financial relationships that could be construed as a potential conflict of interest.
